# High activity Rhenium-186 HEDP with autologous peripheral blood stem cell rescue: a phase I study in progressive hormone refractory prostate cancer metastatic to bone

**DOI:** 10.1038/sj.bjc.6600348

**Published:** 2002-06-05

**Authors:** J M O'Sullivan, V R McCready, G Flux, A R Norman, F M Buffa, S Chittenden, M Guy, K Pomeroy, G Cook, J Gadd, J Treleaven, A Al-Deen, A Horwich, R A Huddart, D P Dearnaley

**Affiliations:** Unit of Academic Radiotherapy and Clinical Oncology, Royal Marsden NHS Trust, Sutton, Surrey SM2 5PT, UK; Department of Nuclear Medicine, Royal Marsden NHS Trust, Sutton, Surrey SM2 5PT, UK; Department of Physics, Royal Marsden NHS Trust, Sutton, Surrey SM2 5PT, UK; Department of Statistics, Royal Marsden NHS Trust, Sutton, Surrey SM2 5PT, UK; Bob Champion Research Unit, Royal Marsden NHS Trust, Sutton, Surrey SM2 5PT, UK; Department of Haematology and Institute of Cancer Research, Royal Marsden NHS Trust, Sutton, Surrey SM2 5PT, UK

**Keywords:** prostate cancer, hormone refractory, radionuclide therapy, peripheral blood peripheral blood stem cell support, rhenium-186 HEDP, palliation, bone metastases

## Abstract

We tested the feasibility and toxicity of high activities Rhenium-186 hydroxyethylidene diphosphonate, with peripheral blood stem cell rescue in patients with progressive hormone refractory prostate cancer metastatic to bone. Twenty-five patients received between 2500 and 5000 MBq of Rhenium-186 hydroxyethylidene diphosphonate followed 14 days later by the return of peripheral blood peripheral blood stem cells. Activity limiting toxicity was defined as grade III haematological toxicity, lasting at least 7 days, or grade IV haematological toxicity of any duration or any serious unexpected toxicity. Activity limiting toxicity occurred in two of six who received activities of 5000 MBq and maximum tolerated activity was defined at this activity level. Prostate specific antigen reductions of 50% or more lasting at least 4 weeks were seen in five of the 25 patients (20%) all of whom received more than 3500 MBq of Rhenium-186 hydroxyethylidene diphosphonate. The actuarial survival at 1 year is 54%. Administered activities of 5000 MBq of Rhenium-186 hydroxyethylidene diphosphonate are feasible using autologous peripheral blood peripheral blood stem cell rescue in patients with progressive hormone refractory prostate cancer metastatic to bone. The main toxicity is thrombocytopaenia, which is short lasting. A statistically significant activity/prostate specific antigen response was seen. We have now commenced a Phase II trial to further evaluate response rates.

*British Journal of Cancer* (2002) **86**, 1715–1720. doi:10.1038/sj.bjc.6600348 www.bjcancer.com

© 2002 Cancer Research UK

## 

Prostate cancer is becoming the most common cause of cancer death in males. Of patients who die from the condition, between 80 and 100% have bone metastases. Bone metastases frequently result in pain, pathological fracture, deformity and neurological deficit and have enormous social and economic implications. Most patients with bone metastases will initially respond to primary androgen deprivation therapy but the duration of this response is variable with a median of 18 months. Once this strategy has failed, second line treatments including corticosteroids, anti-androgens, diethylstilbesterol and cytotoxic chemotherapy including estramustine have only had limited success in preventing disease progression with short-lasting subjective responses in approximately 30% of cases (Dearnaley, 1994; Tannock *et al*, 1996; Pisters, 1999). The average survival in patients with hormone refractory prostate cancer metastatic to bone is in the order of 8 months (Fossa *et al*, 1992; Kelly *et al*, 1993; Sridhara *et al*, 1995; Small and Vogelzang, 1997). Radionuclides including strontium-89, rhenium-186 and samarium-153 have been used in the palliation of bone metastases in prostate cancer (Lewington *et al*, 1991; Porter *et al*, 1993; Quilty *et al*, 1994; Kolesnikov-Gauthier *et al*, 2000). Pain response occurs in about 70% of cases. A correlation between injected activity and rates of pain control in prostate cancer bone metastases have been demonstrated in radionuclide therapy with Strontium-89 (Mertens *et al*, 1993).

Rhenium is a group VII metal with a similar labelling chemistry to Technetium. The radionuclide Rhenium-186 (Re-186) is produced by irradiation of enriched Rhenium-185 and decays with the emission of a β particle (E_max_ 1.07 Mev) and a low abundance (9%) γ ray (137 Kev). Rhenium-186 combined with the bisphosphonate hydroxyethylidene diphosphonate (HEDP) was first suggested as a therapy for prostate cancer bone metastases in the late 1970's (Mathieu *et al*, 1979; Deutsch *et al*, 1986). Since then several investigators have used Re-186 HEDP for the palliation of bone pain in this group of patients with response rates ranging from 30 to 80% with mean duration of response of 7 weeks (Maxon *et al*, 1990, 1991). The activity limiting toxicity has been shown to be thrombocytopaenia with leucopaenia playing a minor role (de Klerk *et al*, 1994a). We postulated that by increasing the administered activity of the radionuclide we can maximise the palliative benefit by delivering ablative doses of ionising radiation to each metastasis. Re-186 HEDP was chosen for a number of reasons. Firstly, it emits β rays, which deliver the therapeutic radiation dose. Secondly, because the radionuclide also emits gamma rays, imaging of the patient is possible, thus providing verification of radionuclide distribution. In order to prevent major bone marrow toxicity especially unrecovered thrombocytopaenia, autologous peripheral blood peripheral blood stem cell rescue (PBSCR) was used in all patients. This technique has been used to protect the bone marrow from the effects of high dose chemotherapy for many years (Mangan, 1995). With a biological half-life of 49–69 h (de Klerk *et al*, 1992) and a physical half-life of 87 h, the effective half-life of Rhenium-186 is approximately 31–38 h. This would lead to <1% activity present in the bone marrow by 9–11 days post therapy and permit peripheral blood stem cell re-14 days after the treatment and before the expected platelet nadir between day 21 and day 28.

A starting activity of 2500 MBq of Re-186 was chosen for the study on the basis of previous work de Klerk *et al* (1994b) and McCready *et al* (1999) which suggested a maximum tolerated activity of between 2500 and 2900 MBq for patients with prostate cancer metastatic to bone without peripheral blood stem cell support. In our own study (McCready *et al*, 1999) two patients treated at activities of 2500 MBq experienced Grade III thrombocytopenia lasting 9 and 11 days.

## MATERIALS AND METHODS

### Eligibility

Eligible patients (Table 1

**Table 1:**
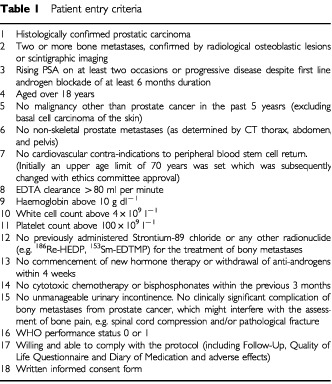
Patient entry criteria

) had progressive hormone refractory prostate cancer metastatic to bone, after at least 6 months first line hormone therapy using androgen suppression. Progressive disease was defined as rising serum Prostate Specific Antigen (PSA) on at least two consecutive readings despite first line androgen blockade, or worsening bone metastases on bone scan, or increasing metastatic bone pain. All men gave written informed consent to take part in the trial which had been approved by the Royal Marsden NHS Trust and Institute of Cancer Research ethics committee.

### Investigation

The full evaluation of patients included full blood count, serum biochemistry including liver function tests, alkaline phosphatase, lactate dehydrogenase, and PSA, isotope (^99m^Tc) bone scans, chest radiograph, CT scan of chest, abdomen and pelvis, ultrasound of kidneys, and bone marrow trephine and aspirate. Quality of Life was assessed using the European Organisation for Research and Treatment of Cancer (EORTC) core Quality of Life Questionnaire version 1 (EORTC/QLQ-C30) (Aaronson *et al*, 1993). This was administered before treatment and subsequently at weekly intervals.

Isotope (^99m^Tc) bone scans were assessed prior to treatment and graded from 1–4 according to severity using the method described by Soloway *et al* (1988). Forty-four per cent (11 out of 25) had a score of 3, 36% (nine out of 25) a score of two and 20% (five out of 25) a score of one.

### Treatment

Peripheral blood stem cell harvesting was performed at least 2 weeks before radionuclide therapy. Patients received 10 μg kg^−1^ of Granulocyte-Colony Stimulating Factor (Filgrastim) for 4 consecutive days and underwent peripheral blood peripheral blood stem cell collection on the fourth and fifth days. We aimed to harvest 1×10^6^ CD34 positive peripheral blood stem cells and 2×10^8^ mononuclear cells per kg of body weight. These were then frozen and retained for use post treatment.

**Figure 1:**
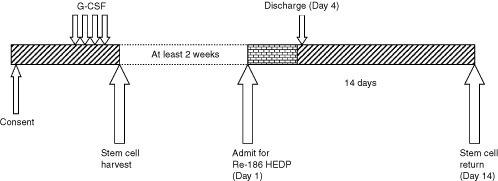
Schema of treatment.

On the day of radionuclide treatment, patients were admitted to a radiation isolation room where intra-venous access was established. A urinary catheter was inserted to minimise contact between radioactive urine and the bladder during the first 24 h. Following instructions to the patient in relevant radioprotection issues the Rhenium-186 HEDP was injected intravenously through the side port of fast running saline infusion. The volume of injected Re-186 HEDP averaged 5 ml. On the day of treatment, Re-186 whole body planar scans were acquired. Full blood counts were measured daily during the admission. In all patients radiation levels decayed to acceptable radioprotection levels by day 4 at which point the patient was discharged. Fourteen days post radionuclide treatment, patients were admitted to a day ward for re-infusion of their peripheral blood stem cells. A graphic illustration of the treatment schema is shown in Figure 1

.

### Follow up and assessment

All patients entered on the study continued their current hormone ablation therapy. Patients were then seen on a weekly basis to monitor their haematological profile, renal function and Quality of Life until the platelet count had recovered which was usually 4 weeks. If grade III haematological toxicity was identified, daily counts were taken until recovery to grade II toxicity or better. Follow-up was then carried out at monthly intervals. A repeat bone scan was performed at 6 weeks post treatment and then on a 3 monthly basis. During the work-up and follow up periods the patients also received best supportive care including external beam radiotherapy and analgesia as required for pain.

### Definition of toxicity

The National Cancer Institute Common toxicity criteria (CTC) version 2 (Trotti *et al*, 2000) was used to define haematological toxicity grades. The activity administered to a patient is a measured known quantity, however the absorbed radiation dose to any point depends on the distribution, elimination and decay of the radionuclide. Activity limiting toxicity (ALT) was defined as grade III or worse platelet toxicity (10 to <50×10^9^ l^−1^) or grade III or worse leucopaenia (1.0 to <2.0×10^9^ l^−1^) of more than 7 days duration, any grade IV haematological toxicity, or any serious unexpected toxicity. Three patients were recruited at each activity level. If no ALT was observed, the next three patients were treated at the next activity level. If one patient had an ALT, the group was expanded to six patients. Maximum tolerated activity was defined if two or more patients out of six reached ALT.

### Statistics

Actuarial survival was calculated using the method of Kaplan and Meier (1958). Correlation between toxicity and activity, performance status, bone scan score, pre-treatment PSA and alkaline phosphatase levels was tested using non-parametric tests (Spearman correlation and Mann–Whitney *U*-tests). The PSA response *versus* administered activity was tested using Fisher's exact test.

## RESULTS

### Patient characteristics

Twenty-five patients were treated with radionuclide therapy. One patient unfortunately developed spinal cord compression after consenting to treatment, but never received Re-186 and is therefore not included in the data analysis. All but one patient had rising PSA levels. This patient had increasing bone pain, worsening bone scan and rising alkaline phosphatase. His serum PSA measured 0.1 ng ml^−1^.

The median time from diagnosis of bone metastases to receipt of Rhenium-186 HEDP was 12 months (range 3–99 months). The median time from primary diagnosis to treatment was 23 months (range 8–123 months). The median age was 64 years (range 50–73) with a median pre-treatment PSA level of 47 ng ml^−1^ (range 0.1–3058) (Table 2

**Table 2:**
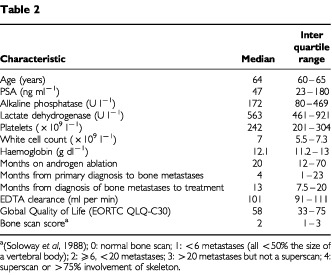

). All patients had been previously treated with lutenising hormone releasing hormone (LHRH) agonists (88%) or orchidectomy (12%). Additionally 84% (21 out of 25) had received prior palliative radiotherapy for painful bone metastases, 80% (20) had received anti-androgens, 16% (four out of 25) were on diethylstilbesterol and one patient had previously received cytotoxic chemotherapy.

The median global Quality of Life score for the group was 58 out of 100 (range 17–100). Bone pain was reported by 80% (20 out of 25) of patients before treatment, with a median EORTC/QLQ-C30 pain measure of 33 out of 100 (range 17–100).

Forty-four per cent (11 out of 25) had a bore scan score of 3, 36% (nine out of 25) a score of 2 and 20% (five out of 25) a score of one. There was excellent correlation between pre-treatment bone scan uptake and the uptake seen on Rhenium-186 planar scans. There was no statistically significant correlation between pre-treatment Soloway score and rate of grade III haematological toxicity. Prescribed activities of Re-186 are shown in Table 3

**Table 3:**
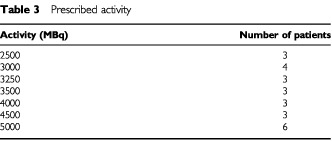
Prescribed activity

. Four patients were treated at the 3000 MBq activity level instead of three. The actual administered activity varied from the prescribed activity on average by 0.12%, range −8% to +8%. The median number of re-infused mononuclear cells per kg was 9.4×10^8^ (range 3.7–21.5). A median of 1.7×10^6^ CD 34 positive cells per kg of body weight (range 0.3–6.7) were re-infused.

### Toxicity

The major toxicity of Re-186 in this study was haematological (Table 4

**Table 4:**
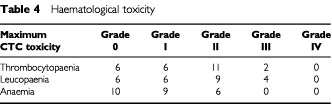
Haematological toxicity

, Figures 2

**Figure 2:**
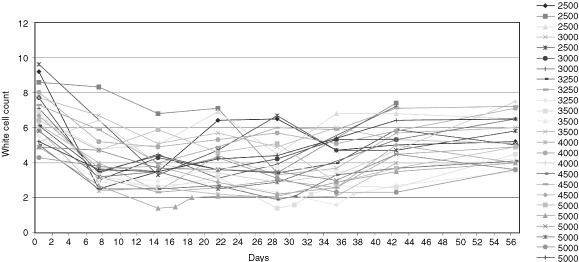
White cell response.

and 3

**Figure 3:**
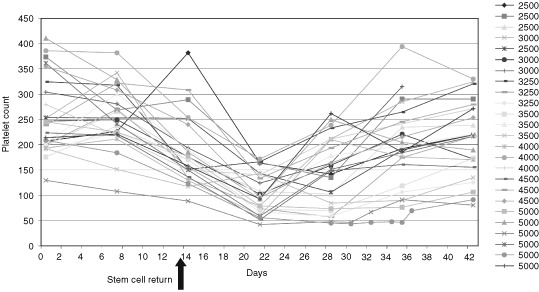
Platelet response.

). One patient out of three in the 5000 MBq activity level experienced grade III thrombocytopaenia lasting 9 days. The patient group at this activity level was therefore expanded to a total of six and a second patient developed grade III thrombocytopaenia of 8 days duration, defining ALT. Grade II thrombocytopaenia occurred in 44% (11 out of 25). Platelet nadir (median 83×10^9^ l^−1^, range 42–172) occurred at a median of 21 days (range 14–28) with a median time from nadir to a count of ⩾150×10^9^ l^−1^ of 7 days (range 7–28).

Grade II leucopaenia was recorded in 36% (nine out of 25). Sixteen per cent of patients (four out of 25) experienced grade III leucopaenia all of who recovered within 7 days and no patients developed neutropaenic sepsis. No grade IV leucopaenia was recorded. The white cell nadir (median 2.95×10^9^, range 1.4–4.9) occurred at a median of 24 days (range 14–35) with a median time from nadir to a count above 3×10^9^ l^−1^ of 14 days (range 14–21). The median nadir haemoglobin level (median 10.7 g l^−1^, range 8.5–13.1), occurred at a median of 20 days (range 7–56). No patient had grade III anaemia, and 24% (six out of 25) had grade II toxicity. Two patients had haemoglobin levels less than 10 g dl^−1^ 7 weeks post treatment, both of who received transfusions. Twenty-four per cent (six out of 25) required transfusion of red cells within 6 months of the therapy however it is unclear whether this is related to the treatment as many of these patients had extensive bone marrow infiltration. There was no non-haematological toxicity noted in the patient cohort.

### Response

Although the primary objective of the study was to establish the maximum tolerated activity of Rhenium-186 HEDP in prostate cancer metastatic to bone, we also recorded PSA and alkaline phosphatase levels and performed follow up bone scans. We looked at PSA response as defined by the recommendations of the PSA Working Group (Bubley *et al*, 1999). A PSA response (⩾50% fall for ⩾4 weeks) was seen in five out of 25 (20%) patients all of whom received activity levels above 3500 MBq. The median duration of response (PSA risen to 50% above nadir level) was 3.5 months. When the presence of a PSA response was analysed according to whether the patient received above or below 3500 MBq, there was a statistically significant PSA activity response seen (*P*=0.015, two-sided Fisher's exact test). A fall in PSA or stable levels was seen in 13 (52%) of patients at 1 month, 11 (44%) at 2 months, and eight (32%) at 3 months. Alkaline phosphatase levels were reduced or stable in 92% (23 out of 25) at 1 month. The number responding reduced to 52% (13 out of 25), and 32% (eight out of 25), at 2 and 3 months respectively with a median decrease of 33%, range 7–76%.

Follow up of pain scores using EORTC QLQ C-30 were available for 23 out of 25 patients. Ten patients had significant pain before treatment as defined by a pain score >17. Of these, eight patients had a pain response as defined by ⩾50% in pain score lasting at least 4 weeks. Global quality of life assessments were available for 23 out of 25 patients. An increase or decrease in the global quality of life score of 25% lasting at least 4 weeks was considered in improvement or deterioration respectively. Eighteen patients had stable global quality of life scores, three of whom demonstrated improvement. Five patients had deterioration in quality of life score.

**Figure 4:**
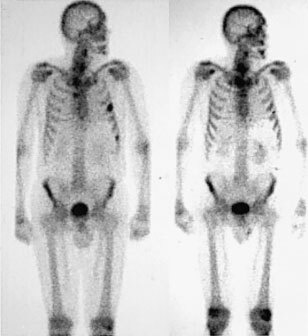
Pre-treatment bone scan (left) showing metastases in left ribs. Bone scan (right) of the same patient 6 months post 5000 MbQ Rhenium-186 HEDP showing disappearance of metastases, correlating with PSA fall from 30 to 0.5 ng ml^−1^.

Follow up ^99m^Tc bones scans were taken between 6 and 12 weeks post treatment. We classified the reports of these scans as stable (no new lesions), improved (reduced number of lesions), or progressive disease (increased number of lesions). Forty-eight per cent (12 out of 25) of scans showed stability or improvement compared to the pre-treatment scans (Figure 4

). The actuarial survival at 1 year is 54% (95% CI 29.5–73.3%) and at 2 years is 23% (95% CI 6–46.6%).

## DISCUSSION

Hormone refractory prostate cancer metastatic to bone is currently incurable. Any useful treatment strategies in this group of patients must aim to produce improvements or stability in quality of life. The main problems experienced by patients with this condition are pain, immobility and lethargy. With a potentially short survival, especially in the progressive and symptomatic disease groups, consideration must be given to the impact of a treatment protocol on patients' lives. This is important with regard to toxicity and time involved in receiving the treatment particularly if it involves inpatient stays. We have demonstrated the feasibility of treating patients with progressive hormone refractory prostate cancer metastatic to bone with escalating activities of Rhenium-186 HEDP using autologous peripheral blood stem cell support. The treatment is well tolerated. Two patients receiving 5000 MBq experienced activity-limiting toxicity in the form of grade III thrombocytopaenia. All patients recovered from grade III haematological toxicity within 10 days. Patients spent a maximum of 4 days as inpatients for radioprotection purposes. Three outpatient day admissions in total were needed to harvest and then re-infuse the peripheral blood stem cells.

PSA responses were seen in five out of 12 patients receiving activities of Re-186 greater than 3500 MBq. No patient had a PSA response below this activity level. This represents a statistically significant activity (dose) response (Table 5

**Table 5:**
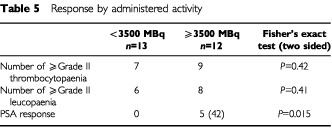
Response by administered activity

). It is unclear whether PSA responses have clinical significance in this group of patients. Some reports have associated PSA response and survival (Sridhara *et al*, 1995; Bubley *et al*, 1999), while others have seen no statistically significant association between palliative benefit and PSA response (Tannock *et al*, 1996).

Having demonstrated feasibility, and established a safe high administered activity level, we now plan to proceed to formally test response to treatment in a phase II single arm study. The main endpoints of this study will be palliative, using quality of life, pain score, and analgesic diaries. However we will also be analysing bone scans for the disappearance of bone metastases and change in biochemical parameters including PSA. Our preliminary assessment suggests 22% of lesions are ablated (McCready *et al*, 1999). A major consideration for the future role of this treatment strategy is the cost benefit. This will be addressed in our phase II trial where the potential benefit of the treatment will be established.

In tandem with our current study we are also investigating methods of dosimetry. Relationships between the administered activity and the dose to bone marrow and to individual metastasis are being studied. Factors involved include body weight, glomerular filtration rate and metastatic burden.

Our hope for the treatment is to prolong symptom free life for patients with hormone refractory prostate cancer metastatic to bone. In theory the radiation dose may be adequate to successfully ablate micrometastases. Bone metastases which elicit enough osteoblastic activity to be detected on bone scanning may be beyond ablation by this method. Recent work (Tu *et al*, 2001) has shown a survival advantage for the addition of strontium-89 to chemotherapy in patients with advanced androgen-independent prostate cancer. We are planning future studies to investigate the combination of chemotherapy with high activity Re-186 HEDP. It is possible that high activity therapy with peripheral blood stem cell rescue using other radionuclides with a short half-life may be valuable both in prostate cancer and other malignancies. We have established that acute toxicity is very acceptable even in this relatively elderly population. Further follow-up and study will be required to find out if there are any more long term effects on bone marrow function from effects on supporting bone marrow stromal cells.
